# Evaluation of periprocedure antibiotics and infection-related hospitalizations after transrectal prostate biopsies

**DOI:** 10.1017/ash.2022.193

**Published:** 2022-05-16

**Authors:** Tenley Ryan, Neena Thomas-Gosain, Jane Eason, Hanna Akalu, Navila Sharif, Jessica Bennett

## Abstract

**Background:** Prostate cancer is the leading cancer diagnosis and the second leading cause of cancer deaths in men. Definitive diagnosis is made by prostate biopsy. This procedure poses a risk of infection and, rarely, sepsis. Studies have found the incidence of symptomatic urinary tract infection (UTI) after biopsy to be 2%–3%, and the rate of infection-related hospitalization (IRH) to be 0.6%–4.1%. An initial review at our facility found the IRH rate to be 3.7%. The primary purpose of this study was to determine the incidence of IRH following prostate biopsy in patients at the Memphis VA Medical Center (VAMC) after initial review and education. **Methods:** All transrectal prostate biopsies performed at the Memphis VAMC from October 2017 through May 2021 were analyzed. Patients were excluded if they had a spinal cord injury or concomitant procedure. The primary outcome was IRH occurring within 30 days of the procedure. Variables collected included risk factors, antibiotic choice and duration, and details of postprocedural infections. Analyses were performed on a per-procedure basis. **Results:** Overall, 601 procedures were identified; 13 were excluded, for a total of 588 transrectal prostate biopsies on 533 patients. All patients were given antibiotics. Oral antibiotics alone were provided for 306 procedures (52%) for an average duration of 3 days. A combination of both oral and intramuscular antibiotics were provided for 282 (48%) procedures. The most common oral antibiotics used were cefuroxime (538, 91.4%), ciprofloxacin (17, 2.9%), amoxicillin–clavulanate (16, 2.7%), and sulfamethoxazole–trimethoprim (12, 2%). Intramuscular antibiotics included ceftriaxone (263, 93.3%) and gentamicin (19, 6.7%). An infectious complication occurred in 29 patients (4.9%): 26 (3.4%) were urogenital and 5 (0.8%) required hospitalization. Of the procedures complicated by a postprocedure infection, 22 (75.9%) received an oral antibiotic alone, 21 (95.4%) of which were cefuroxime, and 7 (24.1%) received both an intramuscular and an oral agent. **Conclusions:** In our initial review, the most common antibiotics used were fluroquinolones, with an average duration of 3 days periprocedure and an IRH rate of 3.7%. These findings were used to reinforce practices compliant with American Urological Association (AUA) guidelines. This follow-up review reveals that the first-line choice changed from fluroquinolones to cephalosporins, with average duration remaining at 3 days. Although the overall infection rate was 4.9%, the IRH rate decreased from 3.7% to 0.8%.

**Funding:** None

**Disclosures:** None

